# From Flames to the Ocean: Biomass Burning Aerosols Are Associated With Changes in Prokaryotic Communities in the Mediterranean Sea

**DOI:** 10.1111/1462-2920.70267

**Published:** 2026-03-30

**Authors:** Nathan Nault, Frédéric Gazeau, Philippe Catala, Barbara Marie, Joan Llort, Cécile Guieu, Matthieu Bressac, Emmanuelle Uher, Maryline Montanes, Elvira Pulido‐Villena, Cristina Santín, Kahina Djaoudi, Pierre E. Galand, Eva Ortega‐Retuerta

**Affiliations:** ^1^ Sorbonne Université, CNRS, Laboratoire d'Océanographie Microbienne (LOMIC), Observatoire Océanologique de Banyuls Banyuls‐sur‐Mer France; ^2^ Sorbonne Université, CNRS, Laboratoire d'Ecogéochimie des Environnements Benthiques (LECOB), Observatoire Océanologique de Banyuls Banyuls‐sur‐Mer France; ^3^ Sorbonne Université, CNRS, Laboratoire d'Océanographie de Villefranche (LOV) Villefranche‐sur‐Mer France; ^4^ Barcelona Supercomputing Center Barcelona Spain; ^5^ IRD, CNRS, Mediterranean Institute of Oceanography (MIO), Aix‐Marseille Université, Université de Toulon Marseille France; ^6^ Biodiversity Research Institute (IMIB), CSIC – Universidad de Oviedo – Principality of Asturias Mieres Spain; ^7^ Biosciences Department Swansea University Swansea UK; ^8^ Septentrion Environnement, Campus Nature Provence Marseille France

**Keywords:** experimental incubations, Mediterranean Sea, prokaryotic abundance, prokaryotic communities, wildfire ash

## Abstract

The Mediterranean basin faces enhanced wildfire risks associated with human‐driven climate and land use changes. Wildfire‐generated aerosols can reach the ocean, where they may subsequently impact marine prokaryotic communities, key drivers of global biogeochemical cycles. However, our understanding of the influence of wildfire airborne particles on the abundance and composition of marine microbes remains limited. We conducted experiments in which surface water from the northwestern Mediterranean Sea was incubated in 300 L minicosms amended with varying amounts of wildfire fine ash particles, previously collected during a Mediterranean wildfire. Wet deposition of wildfire fine ash particles had a short‐term effect on prokaryotes by increasing their abundance and diversity, likely due to the release of both inorganic and organic substrates, alleviating nutrient limitations. Ash deposition could also indirectly affect prokaryotic communities via changes in the composition of phytoplankton populations. These mechanisms induced changes in prokaryotic community composition, reflecting a succession of taxa likely adapted to different substrate qualities. Ash had a negative effect on *Cyanobiaceae* but promoted the growth of *Flavobacteriaceae*, *Rhodobacteraceae* and *SAR11 clade I* among other taxa. Our findings demonstrate that wildfire ash can alter Mediterranean prokaryotic communities during oligotrophic periods, further exacerbating the impact of wildfires on marine ecosystems.

## Graphical abstract

The addition of wildfire fine ash particles (particle size < 20 μm) in Mediterranean summer seawater can increase prokaryotic growth and alter community composition over time, likely through the release of both inorganic and organic substrates by ash particles and by inducing changes in the composition of phytoplankton populations.

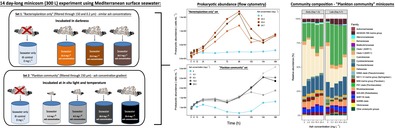

## 1 Introduction

Over recent decades, extreme weather events such as droughts (Mukherjee et al. 2018) and heatwaves (Rossiello and Szema 2019) have intensified worldwide, increasing the risk of extreme wildfires and their potential societal and environmental impacts (Pausas and Keeley 2021; Cunningham et al. 2024). Regions such as the Mediterranean basin, where longer dry seasons and higher temperatures have been reported in recent years (Romano and Ursino 2020), are especially sensitive and considered important fire‐prone areas (Moriondo et al. 2006; Jones et al. 2022). In addition to climate warming, the abandonment of agricultural areas and consequent expansion of forests and shrublands are also increasing the probability of larger and more recurrent wildfires in some parts of the Mediterranean basin (Ascoli et al. 2021; García et al. 2023). The resulting increase in wildfires not only threatens terrestrial ecosystems but also has far‐reaching implications for atmospheric and marine environments. As wildfires occur, they emit aerosols (Wang et al. 2022), which are likely to be deposited in the oceans following atmospheric transport, many times over long‐range distances (Weis et al. 2022). Once deposited, biomass‐burning aerosols enrich surface waters with particles and nutrients, such as phosphorus, nitrogen, trace metals and organic compounds (Guieu et al. 2005; Fujii et al. 2014; Barkley et al. 2019; Ladd et al. 2023), with potential impacts for marine organisms. Indeed, anomalous phytoplankton blooms have been linked to deposition of wildfire aerosols in regions such as the Arctic (Ardyna et al. 2022) or the Southern Ocean (Tang et al. 2021).

Heterotrophic prokaryotes are an important component of oceanic ecosystems, driving essential processes in the cycling of carbon and other elements (Kirchman 2004; Pomeroy et al. 2007). Nearly half of the carbon fixed by primary production in the biosphere is processed by heterotrophic prokaryotes (Azam et al. 1983; Kirchman 2004). Given that the functional contributions of prokaryotic communities are closely tied to their composition (Galand et al. 2018; Morris et al. 2020), understanding the factors that shape their diversity is essential for assessing their impacts on oceanic carbon cycling. In addition to community composition, the efficiency of organic matter processing by heterotrophic prokaryotes depends on the bioavailability and composition of organic matter (Azam and Malfatti 2007; Nagata 2008) as well as inorganic nutrients (Caron 1994; Wambeke et al. 2002). These elements can be found in limiting concentrations in surface oceanic waters (Moore et al. 2013), thereby restricting prokaryotic activity and various metabolic processes. In particular, the Mediterranean Sea exhibits limiting nitrogen and phosphorus concentrations with an overall phosphorus deficit relative to nitrogen, particularly during spring and summer due to water‐column stratification (Pujo‐Pay et al. 2011; Powley et al. 2017). Heterotrophic prokaryotes are usually P‐limited, although they can also experience carbon limitation at various times of the year (Wambeke et al. 2002; Pinhassi et al. 2006; Tsiola et al. 2016).

Recurrent external inputs, such as aerosol deposition (Simoneit 2006; Pulido‐Villena et al. 2008; Djaoudi et al. 2020), can alleviate these limitations and serve as important drivers of prokaryotic carbon remineralisation in the Mediterranean Sea. Several studies have reported increased prokaryotic abundance, activity and community shifts in response to various aerosol types, including Saharan dust (Laghdass et al. 2011; Pulido‐Villena et al. 2014; Guo et al. 2016; Gazeau et al. 2021; Behzad et al. 2022), anthropogenic particles (e.g., traffic‐ and industry‐derived; Marín et al. 2017) and fossil fuel‐derived black‐carbon‐rich aerosols (Weinbauer et al. 2012; Malits et al. 2015; Martinot et al. 2023). For instance, in Mediterranean surface water, *Gammaproteobacteria* and *Bacteroidetes* dominated within two days of exposure to Saharan dust and anthropogenic aerosols (Guo et al. 2016), while *Glacieola* and *Alteromonadaceae* grew following the addition of black‐carbon‐rich material (Weinbauer et al. 2012).

So far, the specific effects of wildfire aerosols on marine prokaryotes have been documented in only three studies worldwide: an 18‐month in situ monitoring study in the Bay of Villefranche‐sur‐Mer (Bonilla‐Findji et al. 2010), a 3‐day‐long experimental approach using Atlantic estuarine water amended with aqueous ash extracts (Gutiérrez‐Barral et al. 2024), and a 5‐day‐long experimental study in which Pacific surface seawater was amended with leachates produced from either natural or lab‐generated ashes (Baetge et al. 2025). These studies reported potential impacts similar to those of other aerosols, including enhanced microbial abundances and community shifts. To the best of our knowledge, no previous study has experimentally investigated the influence of wildfire airborne particle inputs on Mediterranean prokaryotic abundance and community composition. The experimental approach is particularly advantageous, as it allows a focus on the net effects of ash deposition, avoiding the complex interactions between factors inherent in in situ observational studies.

In this study, we aimed to experimentally evaluate the response of a natural Mediterranean Sea prokaryotic community to wildfire fine ash particle exposure. Our initial hypotheses are that (1) ashes stimulate the growth of some prokaryotes in nutrient‐limited Mediterranean summer surface seawater and (2) ash additions lead to community shifts from taxa adapted to oligotrophy to taxa more suited to the utilisation of pulses of nutrients and DOM compounds. To test these hypotheses, a 14‐day‐long experiment was conducted in the northwestern Mediterranean Sea, in which nine 300 L minicosms filled with surface water were amended with varying amounts of wildfire fine ash particles (< 20 μm), as a proxy to airborne particles. The ash was collected after a Mediterranean wildfire in a pine forest in Spain. Prokaryotic abundance was estimated through flow cytometry to investigate changes through time for different quantities of ash, while shifts in community composition were assessed through 16S rRNA metabarcoding.

## 2 Experimental Procedures

### 2.1 Experimental Design

**FIGURE 1:**
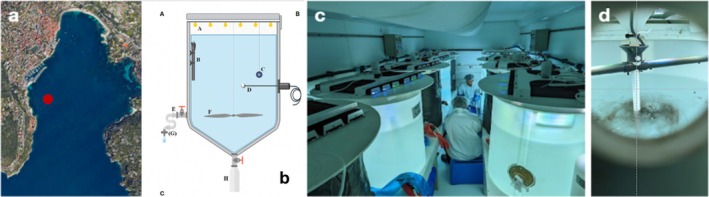
Methods used for the minicosm experiment. (a) Location where the water was pumped from in the bay of Villefranche‐sur‐Mer, France. (b) Scheme illustrating the different components of a minicosm. A: LED cover, B: Heating resistor, C: Temperature data logger, D: Photosynthetically active radiation (PAR) sensor, E: Sampling tube, F: Motorised propeller, G: Filtration cartridge (used only for some parameters) and H: Particle trap. (c) Picture showing eight of the nine minicosms used during the experiment. (d) Picture of the “rain” system taken during the dispersion of ashes at the surface of one of the minicosms.

The experimental facility is the same as the one fully described in Tilliette et al. (2023). Briefly, nine 300 L minicosms, made of trace‐metal‐free high‐density polyethylene (PE), were installed in a light‐isolated, air‐conditioned, clean‐room container. They were equipped with LEDs mimicking the natural light spectrum and intensity as well as with a rotative polyvinyl chloride (PVC) blade generating weak turbulence. The dimensions of the minicosms (1.09 m height, surface area of 0.36 m^2^) allow particles to sink freely within a 1‐m deep layer, offering a realistic contact time between particles and seawater (Figure 1). An orifice positioned at 80 cm below the top of the minicosm allows for easy water sampling. All materials used for the experimental setup and sampling were acid‐washed. Before the onset of the experiment, minicosms were filled with a 2% HCl solution for 1 week and then abundantly rinsed with filtered seawater. They were then directly filled with seawater collected using a high flow peristaltic pump connected to a PE tubing installed at 5 m depth and 200 m from the shore, in the Bay of Villefranche‐sur‐Mer (NW Mediterranean Sea; 43°41.10′N, 7°19.00′E; Figure 1) during July 2022: (i) Five minicosms were filled with seawater passed through a 350 μm mesh (to remove macrozooplankton), later called ‘plankton community’ minicosms; (ii) Four minicosms, later called ‘bacterioplankton only’ minicosms, were filled with 0.2 μm filtered seawater (using Sartorius Sartobran P capsules 0.2 μm). The ‘bacterioplankton only’ were initially designed to quantify the release and dissolution kinetics of nutrients and trace metals from wildfire fine ash particles under abiotic conditions. However, since the filtration step did not completely remove prokaryotes, we used these minicosms to assess prokaryotic responses to wildfire ash exposure in the absence of phytoplankton and in the dark. We acknowledge, however, that the 0.2 μm filtration may have altered the initial microbial community composition by selecting for smaller prokaryotes, thus making direct comparison with the ‘plankton community’ minicosms difficult. The inclusion of a control condition in the bacterioplankton‐only microcosms nevertheless allowed a qualitative assessment of the effects of ash addition on prokaryotic communities in the absence of phytoplankton. All minicosms were incubated for 14 days at constant in situ temperature (20°C). The ‘plankton community’ minicosms were incubated under a simulated diel light cycle at constant intensity (700 μmol m^−2^ s^−1^, 15 h light:9 h darkness), representative of summer surface irradiance in the Mediterranean Sea. In contrast, the ‘bacterioplankton only’ minicosms were incubated in the dark to examine nutrient and trace metals dynamics in the absence of potential light‐dependent biological perturbations (e.g., photosynthesis). The biological parameters presented in this study were sampled during the first 7 days of the experiment. Samples were taken after 1, 6 and 12 h following wildfire ash particle deposition (see next section), and then, on a daily basis from Days 1 to 7, (i) in all minicosms to quantify prokaryotic abundance, and (ii) in ‘plankton community’ minicosms, to assess changes in community composition. Samples for the quantification of autotrophic and heterotrophic prokaryotes abundances were taken in 2 mL cryovials, fixed with glutaraldehyde (0.5% final concentration), flash frozen in liquid nitrogen and stored frozen (−80°C) until analysis. DNA samples were taken only in the ‘plankton community’ minicosms and at six selected time points (24, 48, 72, 96, 120 and 168 h). Although DNA samples were also taken at *T*_0_, they could not be included in the analysis as they were unintentionally lost due to a laboratory handling error. Two extra DNA samples were also taken from 0.1 g of dry ash (< 20 μm size fraction) to identify prokaryotes that could be present, if any. For DNA analyses, 500 mL of seawater was subsampled from the ‘plankton community’ minicosms and filtered through 0.2 μm filters (Whatman Nuclepore). Filters were stored at −80°C until DNA extraction and analysis (within 9 months).

### 2.2 Wildfire Ash Deposition

In this study, wildfire fine ash particles (< 20 μm) were used as a proxy for wildfire airborne particles. Wildfire ash consists of the particulate residue left on the ground after the burning of wildland fuels and includes both mineral materials and charred organic components (Sánchez‐García et al. 2023). Using ash as a proxy is necessary as collecting the amount of wildfire airborne particles needed for an experiment of this scale (up to 40 g after sieving; see below) is not feasible. We chose to use the fraction of ash with a particle size < 20 μm as this was the most similar to aerosols that can be transported over long distances, which was logistically obtainable. However, we acknowledge that this material does not fully represent long‐range transported wildfire particles, which typically fall within a finer size range (usually under 1 μm; Baars et al. 2019; Lu et al. 2025).

The bulk ash sample was collected after a wildfire characterised by a high burn severity (i.e., tree canopy and understory vegetation burnt) in a Mediterranean forest of *Pinus halepensis* in Eastern Spain (40°18′36″ N; 1°01′59″ W; Harper et al. 2019). The bulk ash sample was collected with bare hands, and as its texture was very different from the underlying unburnt soil, it was done in a way that minimised contamination of the sample from the soil. The sample was then air‐dried and stored in the dark in a closed container until use. More details and chemical characterisation of the bulk ash sample can be found in Sánchez‐García et al. (2023) (referred to as SPAE sample in the aforementioned study). The bulk ash was sieved through a 20 μm mesh to select only the finest particles, as previous studies reported a mode at a diameter between 100 and 400 nm for aerosolised wildfire fine ash particles (Lu et al. 2025).

To mimic wet deposition, a system was designed to distribute fine droplets onto the surface of the water in the minicosms. For each seeded minicosm, a solution containing the desired amount of ash was mixed with 1 L of 0.2‐μm‐filtered MilliQ water. This homogeneous solution, continuously agitated on a magnetic stirrer, was directly transported upon mixing in a PE tubing by a peristaltic pump to the water surface. The solution was distributed like rain through holes in a PVC tube parallel to the water surface, which rotated as it was fixed to the axis of the PVC blade, generating weak turbulence in the water (Figure 1). The ‘rain’ lasted for 30 min, which corresponded to the time needed for the complete dispersion of the ash solution, regardless of the ash concentration. Final concentrations of wildfire fine ash particles in the minicosms were 3.3–25.4 mg L^−1^ (Table S1), which correspond to strong but realistic deposition values. They were obtained from CAMS reanalysis data (Inness et al. 2019) for the estimated deposition in the Tasman Sea during the 2019–2020 wildfires in southeastern Australia (Tang et al. 2021) on large Australian wildfires.

Among the four ‘bacterioplankton only’ minicosms, one was used as a control (no ash addition), and for the remaining three minicosms, ~23 mg L^−1^ of ash were dispersed at the surface of each minicosm following the procedure described above (see Table S1 for exact numbers). For the ‘plankton community’ minicosms, a gradient strategy was applied with no ash addition in a control minicosm, while 3.3, 8.5, 18.4 and 25.4 mg L^−1^ of ash were respectively seeded at the surface of the other four minicosms. Due to the limited number of minicosms available, no biological replicates could be included in this experimental setup.

### 2.3 Prokaryotic Abundance

Samples were thawed at room temperature pending measurements. *Synechococcus* and heterotrophic prokaryotes were counted by flow cytometry using a FACSCanto II flow cytometer (Becton Dickinson) equipped with three air‐cooled lasers: blue (argon, 488 nm), red (633 nm) and violet (407 nm). Prokaryotic cells were stained with SYBR Green I (Invitrogen—Molecular Probes) at 0.025% (vol/vol) final concentration for 15 min at room temperature in the dark. Stained prokaryotic cells were discriminated and enumerated according to their right‐angle light scatter (SSC) and green fluorescence measured at 530/30 nm. Photosynthetic and non‐photosynthetic prokaryotes could be distinguished based on their positions in a plot of green versus red fluorescence. *Synechococcus* spp. were then discriminated by their red fluorescence and removed from total prokaryotic counts. *Synechococcus* spp., nano‐ and picoeukaryotes populations were enumerated separately in non‐stained samples according to Marie et al. (2000), discriminated by their strong red and orange fluorescence (585 ± 21 nm). Fluorescent beads (1.002 μm; Polysciences Europe) were systematically added to each sample as an internal standard. The speed of analysis was adapted to cell concentration, usually 1 min at low speed (around 15–20 μL min^−1^) for heterotrophic prokaryotes and 5 min at high speed (around 100 μL min^−1^) for *Synechococcus* sp. populations. The subsequent estimation of cell concentration was determined from the flow rate, which was calculated with TruCount beads (BD Biosciences).

### 2.4 DNA Extraction, 16S rRNA Sequencing and Sequence Analysis

DNA was extracted using the Zymobionics DNA miniprep kit following the manufacturer's instructions with the additional steps of chemical lysis with lysozyme (20 mg mL^−1^) and proteinase K (10 mg mL^−1^). PCR amplification was performed using the universal 16S rRNA primer pair 515F and 926R, covering the V4–V5 hypervariable region (Parada et al. 2016) and sequenced on a MiSeq platform (Illumina) at LGC Genomics GmbH (Berlin, Germany). For the two dry ash samples, DNA extraction, PCR amplification and Illumina sequencing followed the same protocol as for water.

The 16S rRNA amplicons were processed in R (R Core Team 2023) using the RStudio platform (version 2023.12.1.402; Posit team 2024) with the DADA2 pipeline (v1.16.0; Callahan et al. 2016), with the following parameters: truncLen(225, 206), maxN = 0, maxEE = c(2,5) and truncQ = 2. The denoised pair‐end reads were then merged into amplicon sequence variants (ASVs) and filtered for chimaeras using DADA2 denoise‐paired commands. ASVs were taxonomically classified using the SILVA Reference dataset (v138.1; Quast et al. 2012; Yilmaz et al. 2014). Singletons and ASVs assigned to chloroplasts, eukaryotes, and mitochondria were excluded from the dataset. After data cleaning, we obtained an average of 53,262 reads per sample (ranging from 9755 to 103,970) and a total of 573 prokaryotic ASVs.

### 2.5 Statistical Analysis and Data Visualisation

Statistical analyses and data visualisation were all performed in R, under the RStudio environment (version 2023.12.1.402; Posit team 2024) using the packages *vegan* (Oksanen et al. 2022), *Rmisc* (Hope 2022), *corrplot* (Wei and Simko 2021), *zoo* (Zeileis and Grothendieck 2005), *VennDiagram* (Hanbo Chen 2022) and *ggplot* (Wickham 2016). For analyses of diversity and community composition, the number of sequences in each sample was rarefied to the one with the lowest number of reads (*n* = 9755). The Simpson diversity and Pielou's evenness indices, measures of alpha diversity, were calculated.

Sample ordination based on prokaryotic community composition was visualised by Ward's hierarchical cluster analysis based on Bray–Curtis dissimilarity matrix (Murtagh and Legendre 2014; Ricotta and Podani 2017) and non‐metric multidimensional scaling (NMDS). The strength of the NMDS fit was evaluated according to stress value (*S*) (with excellent representation in reduced dimensions for *S* < 0.05, great for *S* < 0.1, good for *S* < 0.2 and poor fitting for *S* > 0.3). Biogeochemical variables (e.g., nutrients, pigments and nature of organic matter; Table S2) were selected following a redundancy analysis to identify and remove redundant variables and then fitted onto the NMDS ordination plots. Details of the measurement procedures for these variables are provided in the Supplementary methods (Supporting Information). Pearson correlations among environmental variables were also calculated. Missing data were replaced by linearly interpolated values of the variables if needed. Differences in community composition per time and ash level were tested with permutational Multianalysis of Variance (PERMANOVA). Analysis of variance (ANOVA) and Tukey's post hoc tests were performed to assess the combined effects of time (early vs. late periods) and ash concentrations on prokaryotic diversity. Homogeneity of variances and normality were tested a priori using Levene's test (homogeneity of variances) and the Shapiro–Wilk test (normality). The significance level (alpha) was set to 0.05. To distinguish between direct and indirect (i.e., phytoplankton response) effects of ash deposition on the prokaryotic community structure, Pearson correlation analyses were carried out between the relative abundance of each ASV, ash concentration and chlorophyll *a* concentration in both early and late periods.

## 3 Results

### 3.1 Changes in Prokaryotic Abundance

**FIGURE 2:**
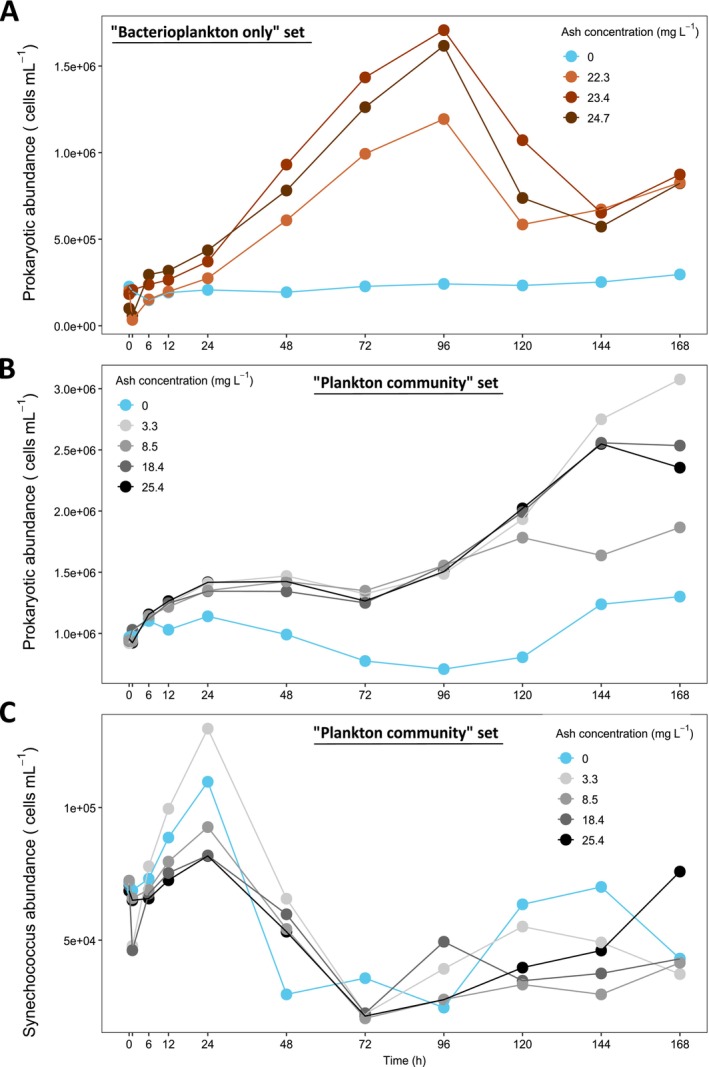
Abundance of heterotrophic prokaryotes through time in the ‘bacterioplankton only’ (A) and ‘plankton community’ (B) minicosms and of *Synechococcus* sp. (C) in ‘plankton community’ minicosms with varying concentrations of ash.

Heterotrophic prokaryotic abundances were always lower in the controls than in all the ash‐amended minicosms (Figure 2A,B). In the ‘bacterioplankton only’ minicosms, despite the filtration step, some prokaryotic cells, with abundances ranging from 1.0 to 2.0 × 10^5^ cells mL^−1^, were observed at the beginning of the incubations. After ash deposition, prokaryotic abundances increased by an order of magnitude in the ash‐amended minicosms, reaching a maximum of 1.7 × 10^6^ cells mL^−1^ at Day 4, before decreasing until Day 6 (144 h), and increasing again on Day 7 (168 h), while the abundances did not vary substantially in the control throughout the experiment (Figure 2A). No phytoplankton cells, including *Synechococcus*, or pico‐ and nanoeukaryotes, were observed in these ‘bacterioplankton only’ minicosms (Figure S1).

In the minicosms containing ash‐amended ‘plankton community’, heterotrophic prokaryotic abundance was 9.5 × 10^5^ cells mL^−1^ before ash deposition (i.e., 6‐fold higher than in the 0.2 um filtered minicosms) and increased 1.5‐fold, reaching 1.5 × 10^6^ cells mL^−1^ 24 h after ash deposition and then plateaued. It increased 72 h after ash deposition, reaching up to 3.0 × 10^6^ cells mL^−1^ by the end of the experiment (Figure 2B). Prokaryotic abundances were similar in all amended treatments for the first 5 days (120 h), but diverged during the second half of the incubations, with the highest abundance observed in the minicosm with an ash concentration of 3.3 mg L^−1^. At the end of the experiment, prokaryotic abundances were 1.4–2.4 times higher in the ash‐amended minicosms compared to the control.

In the ‘plankton community’ minicosms, *Synechococcus* sp. abundances started at 7 × 10^4^ cells mL^−1^ and exhibited a 1.2‐ to 1.8‐fold increase within 24 h following ash deposition in all treatments, reaching abundances up to 1.3 × 10^5^ cells mL^−1^ (Figure 2C). This initial increase was followed by a decline and a subsequent rise after 72 h in all minicosms. The minicosm with an ash concentration of 3.3 mg L^−1^ and the control showed the highest growth compared to treatments with elevated ash concentration after 24 h, with no clear pattern among ash levels at the end of the incubation.

### 3.2 Changes in Prokaryotic Community Composition and Biogeochemical Variables

Prokaryotic communities were separated into three clusters using Ward's hierarchical clustering method based on Bray–Curtis' dissimilarity matrix (Figure S2). The first cluster contained the communities directly extracted from the wildfire fine ash particles, while the other two clusters contained the prokaryotic communities sampled in the minicosms. The ASVs from the dry ash aliquots (142 ASVs) were absent or only found at very low abundance (< 30 reads) in the ash‐amended minicosms samples (Table S3). All the most abundant ASVs in the dry‐ash aliquots have been previously identified as potential contaminants of laboratory reagents (Salter et al. 2014), suggesting that none, or only a few, prokaryotes were directly present in the ash. Therefore, it was decided to remove these ASVs only observed in the dry ash aliquots from the subsequent analyses.

**FIGURE 3:**
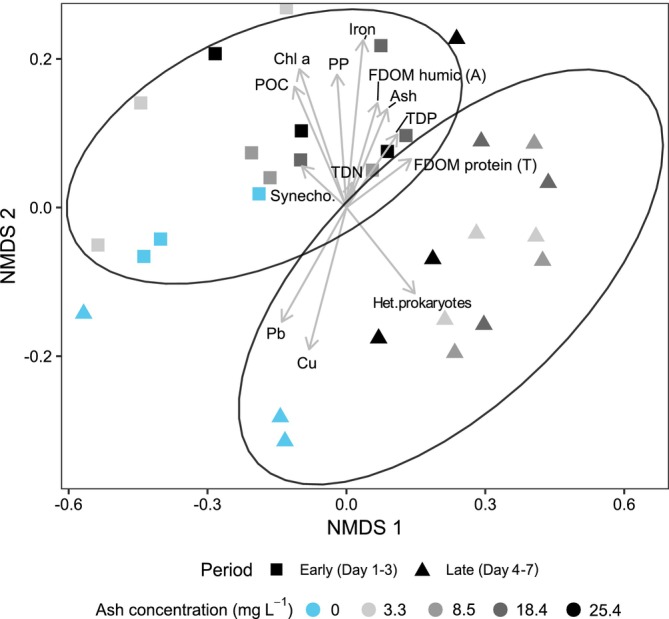
Non‐metric multidimensional scaling (NMDS) of prokaryotic community composition in ‘plankton community’ minicosms amended with different quantities of ash (stress: 0.1). Colours represent the ash concentration, shapes represent the incubation period (squares = early, triangles = late). Black ellipses around the centroids indicate communities identified as significantly different by PERMANOVA analysis. Arrows correspond to biogeochemical variables. Ash = ash concentration; Het.prokaryotes = total prokaryotic abundance; Synecho. = *Synechococcus* sp. abundance; Chl a = chlorophyll *a* concentration; TDN = total dissolved nitrogen concentration; TDP = total dissolved phosphorus concentration; POC = particulate organic carbon concentration; PP = particulate phosphorus; Iron = dissolved iron; Pb = dissolved lead; Cu = dissolved copper; Fluorescent Dissolved Organic Matter (FDOM); humic (A) = Humic‐like fluorescent dissolved organic matter (A peak); Fluorescent Dissolved Organic Matter (FDOM); protein (T) = protein‐like fluorescent dissolved organic matter (T peak).

Based on their prokaryotic community structure, samples were separated into two main groups (PERMANOVA, *p* = 0.001, Figure 3). The first group contained samples taken from Days 1 to 3 (later referred to as ‘early’ samples), while the second one contained samples from Days 4 to 7 (hereafter ‘late’ samples). Based on this, the further analysis was split into these two groups. Additionally, a distinction between samples from the control and ash‐amended minicosms was observed, particularly during the late period (Figure 3).

Chlorophyll *a*, particulate matter and dissolved nutrients (e.g., trace metals, humic‐like fluorescent dissolved organic matter and phosphorus) were mostly clustered with ash‐amended samples from the early period (especially after 24 and 48 h; Figure 3). The variables showing the highest Pearson correlation values to ash concentration were Humic‐ and Protein‐like Fluorescent Dissolved Organic Matter (FDOM) (0.99 and 0.93, respectively, *p* < 0.001), total dissolved phosphorus (0.96, *p* < 0.001) and dissolved iron (0.68, *p* < 0.001) concentrations. In the late period, the amended samples were characterised by a high abundance of heterotrophic prokaryotes and protein‐like fluorescent dissolved organic matter, while the controls were linked to dissolved lead and copper concentrations.

### 3.3 Changes in Prokaryotic Diversity

**FIGURE 4:**
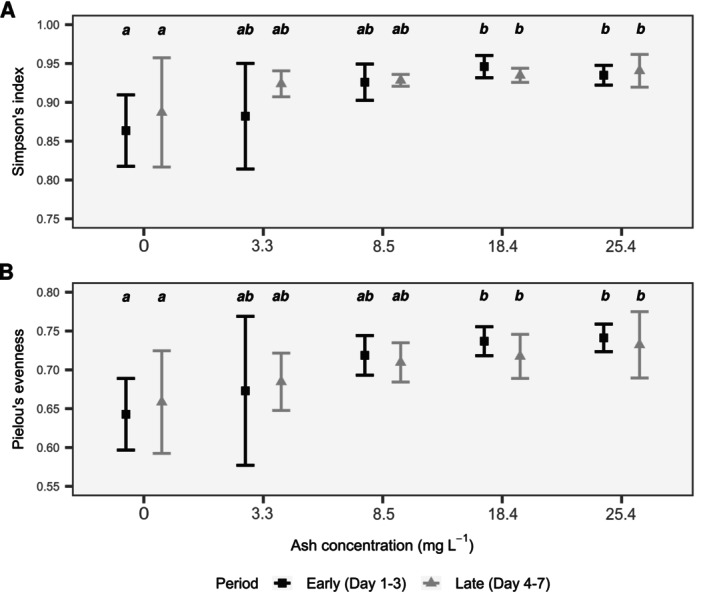
Diversity (mean ± SD) of the prokaryotic communities measured with the Simpson's index (A) and Pielou's evenness (B) for each level of ash deposition in early (black squares) and late (grey triangles) incubation periods. The bars represent the standard deviation of the data. Different letters indicate significantly different groups (Tukey's post hoc test, *p* = 0.05). Groups sharing a letter do not differ significantly.

For both the early and late periods, prokaryotic diversity, measured as Simpson's or Pielou's index, significantly increased with ash deposition (two‐way ANOVA, *p* = 0.018 and *p* = 0.008 for Simpson and Pielou, respectively) but did not differ between the two periods (two‐way ANOVA, *p* = 0.31 and *p* = 0.93 for Simpson and Pielou, respectively) (Figure 4). Compared to the control, prokaryotic diversity (Simpson and Pielou) was significantly higher (1.1‐fold) for ash concentrations of 18.4 mg L^−1^ (Tukey's HSD, *p* = 0.027 and *p* = 0.038 for Simpson and Pielou, respectively) and 25.4 mg L^−1^ (Tukey's HSD, *p* = 0.037 and *p* = 0.016 for Simpson and Pielou, respectively). Diversity values at the two highest concentration levels were nearly identical and did not differ significantly from each other.

### 3.4 Changes in Prokaryotic Taxonomic Composition

**FIGURE 5:**
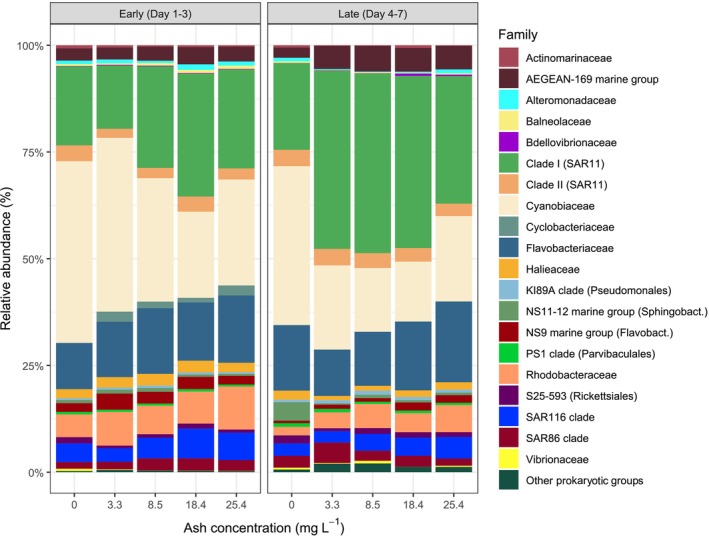
Average relative abundances of the most abundant prokaryotic families (> 1% in at least one sample) in each experimental condition of the ‘plankton community’ minicosms, in the control and after ash deposition in the early and late incubation periods. Other prokaryotic groups represent the ASVs that were not assigned at the family level.

The addition of ashes promoted a succession in the prokaryotic community as 79 ASVs were found exclusively in ash‐amended minicosms in the early period and 92 ASVs in the late period, while only 16 and 54 ASVs were exclusive to the controls in the early and the late periods, respectively (Figure S3). In early samples after ash deposition, *Cyanobiaceae* (30.8% ± 9.6% on average), *SAR11* (*clade I*) (21.4% ± 5.3% on average), and *Flavobacteriaceae* (13.5% ± 2.0% on average) dominated prokaryotic communities at the family level (Figure 5). The dominance of these families varied between the different levels of ash deposition. When ash deposition was higher, the relative abundance of *Cyanobiaceae* decreased, while *SAR11* (*clade I*), *Flavobacteriaceae*, *Rhodobacteraceae*, *SAR116 clade* and *Cyclobacteriaceae* increased (Figure 5).

In the late period, prokaryotic communities were dominated by the same families as in the early samples, although *SAR11* (*clade I*) (34% ± 9.5%) were relatively more abundant than *Cyanobiaceae* (20.5% ± 8.9%) and *Flavobacteriaceae* (14.4% ± 3%) (Figure 5). In the control treatment, we observed the presence of the *NS11‐12 marine group* family (4.2% ± 2.2%) and the dominance of *Cyanobiaceae* (35.8% ± 16.5%), which changed to a dominance of *SAR11* (*clade I*) and an increase of *Flavobacteriaceae*, *Rhodobacteraceae* and *SAR116 clade* in ash‐amended samples. The relative abundance of the *AEGEAN169 marine group* family was twofold higher in the ash‐amended (5.5% ± 1.8%, on average) than in the control (2.3% ± 1.1% on average) minicosm.

**FIGURE 6:**
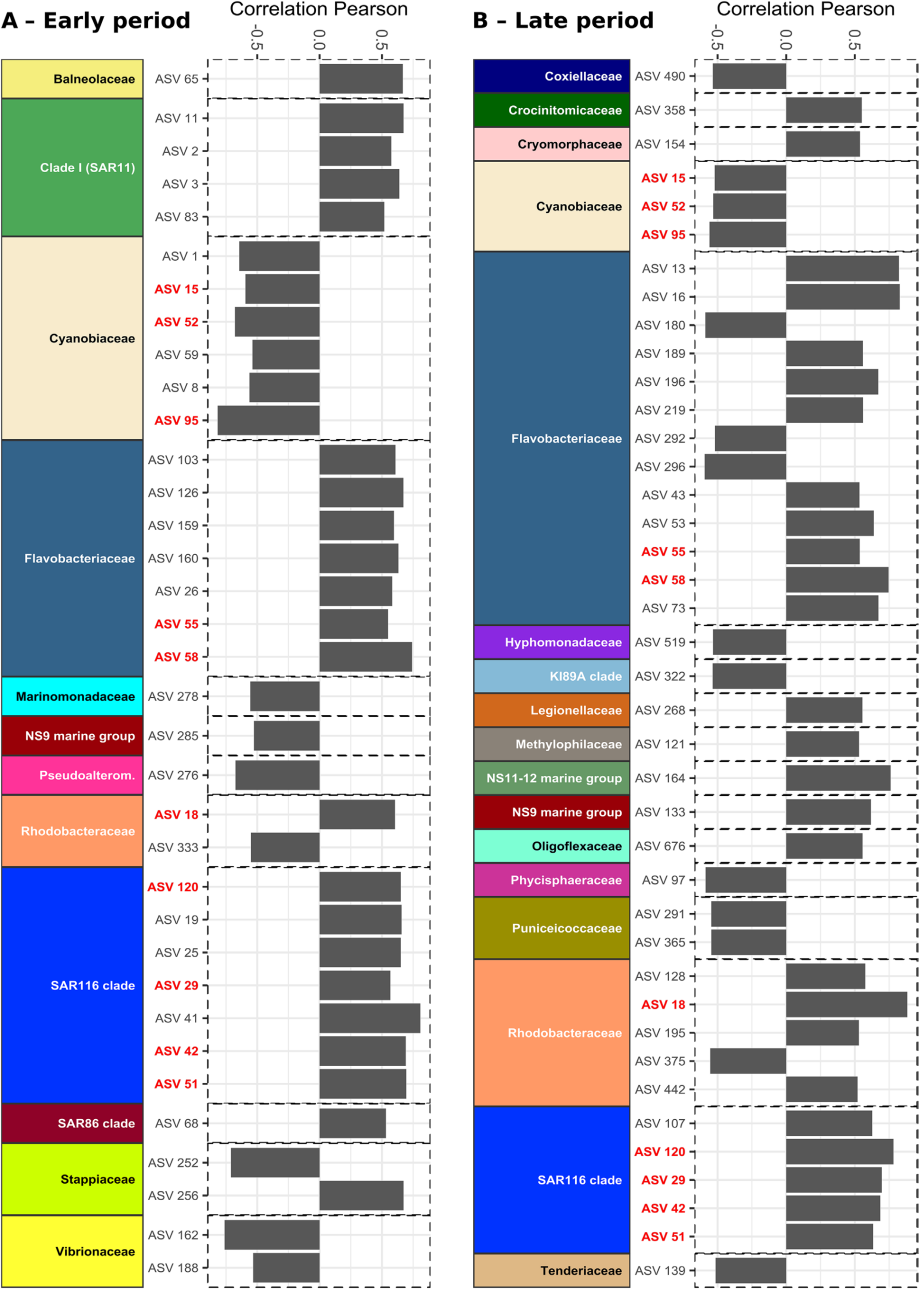
Prokaryotic ASVs whose relative abundance is correlated to ash concentration during (A) the early and (B) the late incubation period. Colour bars on the left represent the families where these ASVs belong (colours are as presented in Figure 5). Grey bars represent the value of the Pearson correlation coefficient. ASVs correlated to ash in both early and late periods are identified in red.

**FIGURE 7:**
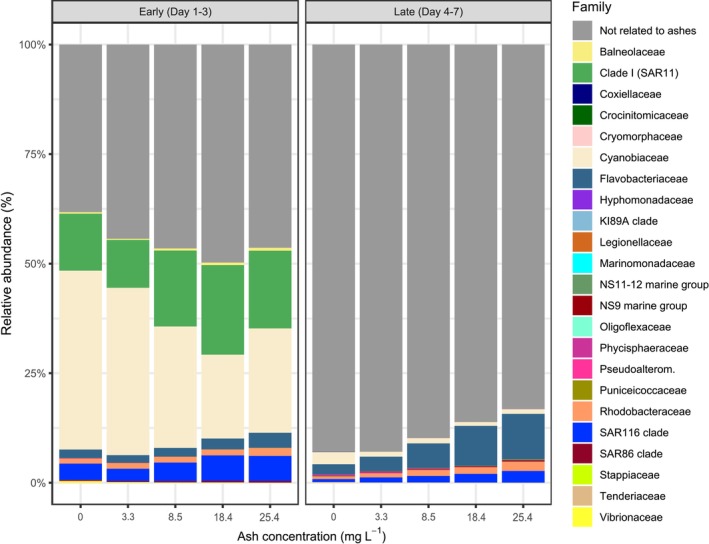
Relative abundances of the prokaryotic ASVs significantly correlated with ash concentration, grouped by family, across experimental conditions during the early (left panel) and the late (right panel) periods of the experiment. ‘Not related to ashes’ refers to all ASVs that were not identified as significantly correlated to the ash concentration.

We conducted correlation analyses between the relative abundance of each ASV in the early or late samples and ash deposition (*n* = 15 for each correlation analysis). In the early period, 35 out of 573 ASVs, belonging to 12 different families, were significantly correlated to ash concentration (Figure 6) and represented on average 54.9% ± 4.3% of total sequences (Figure 7). The identified ASVs belonged mostly to the families *Cyanobiaceae* (negative correlations), *Flavobacteriaceae, SAR116* and *SAR11* (*clade I*) ASVs (positive correlations). Among the less represented families, some presented negative (e.g., *Vibrionaceae*) or positive (e.g., *SAR86*) responses to the ash deposition.

In the late period, 40 out of 573 ASVs, belonging to 17 families, were significantly correlated to ash concentration, but they only represented 11.1% ± 4.4% of total sequences on average (Figures 6 and 7). *Flavobacteriaceae* showed the highest number of ASVs (13) correlated to ash, followed by *Rhodobacteraceae* (5), *SAR116 clade* (5), all showing positive correlations, and *Cyanobiaceae* (3), which showed negative correlations with ash concentration (Figure 6). Among ASVs belonging to less frequent families, there were both positive (e.g., *NS9 marine group*) and negative (e.g., *Puniceicoccaceae*) correlations to ash deposition.

A total of 10 ASVs, belonging to four different families (e.g., *Flavobacteriaceae*), were significantly correlated with ash concentration in both the early and late periods (Figure 6). Some families were exclusively found to be correlated to ash concentration in the early (e.g., *SAR11* (*clade I*)) or in the late period (e.g., *NS11‐12 marine group*).

For both periods, ASVs correlated to chlorophyll *a* represented no more than 20% of the sequences, suggesting that phytoplankton (at least with chlorophyll *a* as a surrogate) was not a main driver of prokaryotic community structure (Figure S4).

## 4 Discussion

This experimental study demonstrates that the deposition of wildfire fine ash particles, used as a proxy for wildfire aerosol particles, impacts prokaryotic communities in the Mediterranean Sea, both through the stimulation of microbial biomass build‐up and through changes in diversity and community composition. The observed increase in prokaryotic abundance and changes in composition after amendments with wildfire fine ash particles could be due to the addition of prokaryotic cells present in the ash, or to the stimulation of marine prokaryotic communities by substances released by the ash. We ruled out the first option since the few ASVs found in the ash were not present in the minicosms. However, it is important to recognise that, in nature, microbial cells can be transported alongside wildfire airborne particles (Moore et al. 2021; Kobziar et al. 2022) and deposited in the ocean.

Our results showed that wildfire ash deposition induced a two‐step response in prokaryotic abundance. An immediate response observed in the ‘bacterioplankton only’ minicosms was likely associated with the release of substrates by the ash itself rather than from phytoplankton, which were absent from these incubations. Dissolved phosphorus, micronutrients and organic matter, with a lower release of nitrogen (data not shown), partially alleviated the nutrient limitation encountered in the Mediterranean Sea during summer (Pujo‐Pay et al. 2011; Powley et al. 2017). Although the experimental conditions differed between the ‘bacterioplankton‐only’ and ‘plankton community’ incubations, we hypothesise that the rapid prokaryotic response observed in the plankton community minicosms is at least partly attributable to a direct effect of ash addition. Ash is recognised as a source of partially bioavailable inorganic nutrients and organic matter (Perron et al. 2022; Sánchez‐García et al. 2023; Baetge et al. 2025), thereby likely promoting prokaryotic growth. The magnitude of ash‐driven effects on prokaryotic growth depends on initial abundance (the lower it is, the stronger the response is observed, Baetge et al. (2025)). This observation is consistent with our results, which showed a higher increase of prokaryotic abundance during the first 48 h in the ‘bacterioplankton only’ minicosms (~5‐fold), compared to the ‘plankton community’ minicosms (~0.7‐fold). Further experiments are, however, needed to compare ash effects with and without the presence of phytoplankton under similar experimental conditions.

Although this study is the first to experimentally address the effects of wildfire ash on Mediterranean Sea microbial communities, previous studies reported similar quick increases in microbial abundances following nutrient‐rich dust deposition (Lekunberri et al. 2010; Pitta et al. 2017; Gazeau et al. 2021), addition of fossil fuel pyrolysis products (Martinot et al. 2023) or wildfire‐derived compounds (González‐Sánchez et al. 2024) in stratified Mediterranean Sea waters. Our results also align with previous observations in Mediterranean (in situ), Atlantic, and Pacific (experimental) surface seawater, which showed a 1.5‐ to 2‐fold increase in prokaryotic abundances (comparable to the increase observed here in the ‘plankton community’ minicosms), and alterations of the community composition within 3 days following exposure to wildfire ash (Bonilla‐Findji et al. 2010; Gutiérrez‐Barral et al. 2024; Baetge et al. 2025). Collectively, these results confirm that exposure to airborne particulate matter or aerosol‐derived compounds, irrespective of their origin, can induce the growth of marine prokaryotic communities.

Unlike the bacterioplankton‐only minicosms, ash deposition induced a secondary response in prokaryotic growth after 72 h only in all ash‐amended ‘plankton community’ minicosms. Because higher ash concentrations coincided with peaks in chlorophyll *a* after 24 h, we hypothesise that prokaryotes in the ‘plankton community’ minicosms were responding to changes in the phytoplankton community. These changes, reflected by changes in pigment concentrations, primarily those associated with diatoms and dinoflagellates (Figure S5), and the subsequent decline in phytoplankton pigments, may have promoted the release of labile substrates used by prokaryotes (Obernosterer and Herndl 1995; Thornton 2014). Together, these results highlight that the effects of wildfire ash deposition may have both direct and indirect effects on prokaryotic communities, depending on the response of phytoplankton cells.

We also observed an increase in prokaryotic diversity in response to increasing ash concentrations, which suggests that the deposition of wildfire fine ash particles promoted the growth of multiple taxonomic groups rather than favouring a few dominant species. Our results may be unexpected as substrate inputs usually lead to the dominance of copiotrophic taxa and a subsequent decrease of prokaryotic diversity (Mou et al. 2008; Teeling et al. 2012). For instance, Zhang et al. (2017) showed decreases in prokaryotic diversity after the exposure of western Pacific surface seawater to volcanic ash, which induced a dominance of *Rhodobacteraceae* and *Alteromonadaceae*. However, increases in microbial diversity following sandstorms have also been reported in the Red Sea (Behzad et al. 2022). The dust deposition associated with sandstorms resulted in large nutrient, organic matter and particle inputs, which likely supported the growth of more taxa. Similarly, it was hypothesised that a high diversity of DOM compounds is likely to enhance prokaryotic diversity (Farjalla et al. 2009). The ash introduced released micro and macronutrients in the minicosms, but the particles themselves could also have provided new surfaces for microbial attachment with probable biofilm development, facilitating niche diversification (Mestre et al. 2017). Although not quantifiable, this hypothesis is supported by our cytogram results, showing dots exhibiting green fluorescence and with higher side scattering (surrogate of size) than single cells, which could be ash particles with attached stained bacteria (Figure S6). As a result, higher ash quantities may have further enhanced these effects, allowing more taxa to access substrates and contributing to the observed increase in diversity. Collectively, these results suggest that the effects of exposure to aerosols on prokaryotic diversity vary with local conditions and/or the composition, concentration and nature of aerosols. However, the weaker increase in diversity observed at the highest ash concentrations suggests that the community may have reached a saturation point, where further inputs no longer translate into diversity increase. This saturation likely reflects environmental constraints, such as intensified interspecific interactions, physical or resource limitations (Loreau 2000).

The shift in prokaryotic community structure observed 3 days after the ash deposition in all treatments marked a clear difference between the early (Days 1–3) and late (Days 4–7) periods of incubation. As mentioned above, these two periods were also contrasted in terms of phytoplankton pigments. We can thus hypothesise that changes in prokaryotic community structure were related to changes in phytoplankton‐derived substrate composition over time. Our results also confirm previous studies that observed shifts in community composition after 48 h of exposure to leachates produced from forest fire ashes (Gutiérrez‐Barral et al. 2024), and 3 h after the addition of Saharan dust to Mediterranean waters (Guo et al. 2016). However, our experimental design does not allow us to disentangle the extent to which prokaryotic responses are driven directly by the input of wildfire fine ash particles versus indirectly by ash‐stimulated phytoplankton growth and the resulting changes in DOM composition.

*Alphaproteobacteria*, *Bacteroidia* and *Cyanobacteriia* were the dominant prokaryotic classes during the early and late periods of the experiment in both control and ash‐amended minicosms, which are frequently observed in the surface Mediterranean waters (Schauer et al. 2003; Lambert et al. 2019). Within these classes, specific families (*Cyanobiaceae*, *Flavobacteriaceae*, *Rhodobacteraceae*, *SAR11 (clade I)* and *SAR116 clade*) exhibited notable changes in relative abundance between the different ash concentrations. For *Cyanobiaceae*, the lack of a consistent pattern in *Synechococcus*' abundance across ash concentrations (flow cytometry) suggests that the initial increase was not driven by ash deposition. Their relative abundance based on 16S rRNA sequencing was negatively correlated with ash input, indicating a detrimental effect of ash on *Cyanobiaceae* growth. This is likely due to large phytoplankton cells outcompeting cyanobacteria, which are better suited for nutrient‐poor environments (Scanlan 2003) and could have slower growth rates than microphytoplankton, especially in nutrient‐enriched environments. Supporting this hypothesis, we observed a prevalence of pigments belonging to larger phytoplankton groups (diatoms and dinoflagellates) when ashes were added (Figure S5b,d). In addition, Sisma‐Ventura and Rahav (2019) also showed that heterotrophic prokaryotes are able to outcompete autotrophic microbes in the presence of elevated phosphorus in the Mediterranean Sea during summer, suggesting that the observed decrease of *Cyanobiaceae* could Falso be linked to competition with heterotrophic prokaryotes for access to substrates.

The *SAR11* (*clade I*) proportion increased in the ash‐amended minicosms compared to the control during both periods. Our results contrast with earlier findings showing declines in *SAR11*'s relative abundance in plankton communities during perturbation experiments, such as substrate amendments (Giovannoni 2017), likely due to its weaker competitive abilities in nutrient‐rich environments (Guo et al. 2016). However, *SAR11*'s low carbon and nitrogen content relative to phosphorus (White et al. 2019) might give them an advantage in our study's phosphorus‐enriched (or rather nitrogen‐deficient) environment. *SAR11* are also known users of low‐molecular‐weight and labile compounds (Malmstrom et al. 2005; Giovannoni 2017). This suggests that the increase in their relative abundance could be linked to an increase in the availability of these substrates in ash‐amended minicosms, either through direct leaching from the ashes or degradation of complex compounds by free‐living or particle‐attached prokaryotic groups (e.g., *Flavobacteriaceae*), resulting in higher *SAR11* growth. Our experimental setup with large volume minicosms might also have favoured the growth of *SAR11* by reducing the drawbacks of using a closed system (e.g., lack of supply of fresh organic matter, nutrients and bacteria, accumulation of toxic metabolites), known as the ‘bottle effect’ (Ionescu et al. 2015), which has been reported to negatively affect the growth of *SAR11* (Haro‐Moreno et al. 2019).

Other taxa, such as *Bacteroidia* (*Flavobacteriaceae* and *Cyclobacteriaceae*) and *Rhodobacteraceae*, exhibited higher relative proportions in ash‐amended minicosms, likely reflecting a rapid response (< 72 h) to the ash deposition. These families are known to metabolise various complex substrates, including phytoplankton‐derived DOM (Teeling et al. 2012; Taylor and Cunliffe 2017; Borchardt et al. 2020; Behzad et al. 2022), humic‐like DOM (*Rhodobacteraceae*; Bouchachi et al. 2023), terrestrial DOM (*Bacteroidia*; Zhao et al. 2023) and wildfire‐derived substrates (*Rhodobacteraceae*; González‐Sánchez et al. 2024).

Additionally, *Rhodobacteraceae*, which can colonise surfaces in aquatic environments (Taylor and Cunliffe 2017), may have used ash particles both as substrate sources and attachment sites (Bischoff et al. 2021). *Flavobacteriaceae* can metabolise organic pyrolysis byproducts such as naphthalene (Galachyants et al. 2020), suggesting that this ability could have contributed to their growth, especially in the early period.

Members of the *SAR116 clade* were also positively responding to ash deposition during both periods, suggesting that the amendment benefited this family either directly or indirectly. Interestingly, these ASVs were correlated to ash concentration during both periods, suggesting that they were benefiting from the ash inputs throughout the experiment, or from both ash and phytoplanktonic DOM. The *SAR116 clade* is primarily known to rely on simple and labile organic compounds (Sosa et al. 2019), and its rapid response to ash deposition in the early period suggests that such compounds might have been introduced by the ash. However, it is also possible that other bacterial groups, such as *Bacteroidetes*, played a key role by breaking down complex DOM from either ash or phytoplankton into simpler molecules throughout the experiment (Behzad et al. 2022), hence supporting the growth of other bacterial clades such as *SAR116*. In addition, the known motility of *SAR116* may have allowed it to colonise particles (Sosa et al. 2019).

## 5 Conclusion and Perspectives

Our findings demonstrated that wildfire fine ash particles, a proxy of wildfire airborne particles, affect prokaryotic communities in the oligotrophic Mediterranean Sea by stimulating both their abundance and diversity, and by changing their composition. These effects likely result from a combination of direct effects, via the supply of both inorganic and organic substrates, and indirect effects through the change in pigment concentration, which may reflect shifts in phytoplankton community structure and, consequently, in the composition of organic substrates for prokaryotes. We saw significant shifts in microbial community composition over time, reflecting the natural microbial succession likely driven by changes in available substrates and substrate preferences throughout the experiment. Ash amendment reduced the proportion of *Cyanobiaceae*, likely due to their reduced suitability and competitive disadvantage with respect to other phytoplankton groups in substrate‐rich environments, and increased the proportion of families like *Flavobacteriaceae*, *Rhodobacteraceae* and the *SAR11 clade I*. While this study provides an initial assessment of the responses of Mediterranean prokaryotic communities following exposure to wildfire ash, these findings may also be relevant to other marine environments subject to wildfire aerosol deposition, offering essential insights into the potential impacts of these hitherto little‐studied phenomena on marine ecosystem functioning and biogeochemical cycles in low‐nutrient oceanic regions where prokaryotes play crucial roles. Further work should aim to resolve the full sequence linking changes in phytoplankton dynamics, DOM composition, and prokaryotic communities in response to biomass burning aerosol deposition. Ash addition experiments under similar environmental conditions, conducted both in the presence and absence of phytoplankton, would help distinguish the direct and indirect effects of wildfire fine ash particles. Additionally, exposing prokaryotic communities separately to ash leachates and to ash particles devoid of soluble compounds, while differentiating between particle‐attached and free‐living prokaryotes, would allow the separation of effects driven by the physical presence of ash from those mediated by its chemical release.

## Author Contributions

**Nathan Nault:** data curation, formal analysis, data visualisation, writing – original draft, writing – review and editing. **Frédéric Gazeau:** conceptualization, methodology, investigation, writing – review and editing. **Philippe Catala:** investigation, writing – review and editing. **Barbara Marie:** investigation. **Joan Llort:** conceptualization, methodology, investigation, writing – review and editing, funding acquisition. **Cécile Guieu:** conceptualization, methodology, investigation, writing – review and editing, funding acquisition. **Matthieu Bressac:** conceptualization, methodology, investigation, writing – review and editing. **Emmanuelle Uher:** methodology, investigation, writing – review and editing. **Maryline Montanes:** methodology, investigation, writing – review and editing. **Elvira Pulido‐Villena:** methodology, investigation, writing – review and editing. **Cristina Santín:** methodology, writing – review and editing. **Kahina Djaoudi:** methodology, investigation, writing – review and editing. **Pierre E. Galand:** writing – original draft, writing – review and editing, supervision, validation. **Eva Ortega‐Retuerta:** conceptualization, writing – original draft, writing – review and editing, supervision, validation, funding acquisition.

## Funding

This work was supported by the Institut national des sciences de l'Univers, the European Space Agency amd Agence Nationale de la Recherche (ANR‐10‐INBS‐0, ANR‐21‐ESRE‐0038).

## Conflicts of Interest

The authors declare no conflicts of interest.

## Supporting information


**Figure S1:** Flow cytometry Synechococcus, nanoeukaryotes and picoeukaryotes in the “bacterioplankton only” minicosms ‐ from flames to the ocean Nault 2026.
**Figure S2:** Dendrogram based on prokaryotic community composition ‐ from flames to the ocean Nault 2026.
**Figure S3:** Venn diagram ‐ from flames to the ocean Nault 2026.
**Figure S4:** Relative abundances of the prokaryotic ASVs significantly correlated with chlorophyll a levels ‐ from flames to the ocean Nault 2026.
**Figure S5:** Pigments concentration in the “plankton community” minicosms ‐ from flames to the ocean Nault 2026.
**Figure S6:** Cytogram examples ‐ from flames to the ocean Nault 2026.

**Table S1:** Ash deposition and fluxes.

**Table S2:** List of biogeochemical and biological variables.

**Table S3:** ASVs in dry ash aliquots.

## Data Availability

The data that support the findings of this study are openly available in the European Nucleotide Archive at https://www.ebi.ac.uk/ena/submit/webin/report/runs/ERP165898, reference number PRJEB82131.
