# Supplementary material for: From Flames to the Ocean: Biomass Burning Aerosols Are Associated With Changes in Prokaryotic Communities in the Mediterranean Sea
Source: Environ Microbiol. 2026 Mar 30;28(4):e70267. doi: 10.1111/1462-2920.70267 (PMC13036370; doi:10.1111/1462-2920.70267)
Supplement: Supplementary file 1 — Figure S1: Flow cytometry Synechococcus, nanoeukaryotes and picoeukaryotes in the “bacterioplankton only” minicosms ‐ from flames to the ocean Nault 2026. Figure S2: Dendrogram based on prokaryotic community composition ‐ from flames to the ocean Nault 2026. Figure S3: Venn diagram ‐ from flames to the ocean Nault 2026. Figure S4: Relative abundances of the prokaryotic ASVs significantly correlated with chlorophyll a levels ‐ from flames to the ocean Nault 2026. Figure S5: Pigments concentration in the “plankton community” minicosms ‐ from flames to the ocean Nault 2026. Figure S6: Cytogram examples ‐ from flames to the ocean Nault 2026. [file EMI-28-e70267-s003.docx]

**Supplementary material – From flames to the ocean: biomass burning aerosols are associated with changes in prokaryotic communities in the Mediterranean Sea**

**Nathan Nault**^1,2^ ([nault@obs-banyuls.fr](mailto:nault@obs-banyuls.fr); ORCID: 0009-0000-5072-7652), Frédéric Gazeau^3^ ([frederic.gazeau@imev-mer.fr](mailto:frederic.gazeau@imev-mer.fr); ORCID: 0000-0001-8807-4597), Philippe Catala^1^ ([catala@obs-banyuls.fr](mailto:catala@obs-banyuls.fr); ORCID: 0009-0006-8262-5275), Barbara Marie^1^ ([marie@obs-banyuls.fr](mailto:marie@obs-banyuls.fr); ORCID: 0000-0003-0005-6365), Joan Llort^4^ ([joan.llort@bsc.es](mailto:joan.llort@bsc.es); ORCID: 0000-0003-1490-4521), Cécile Guieu^3^ ([cecile.guieu@imev-mer.fr](mailto:cecile.guieu@imev-mer.fr), ORCID: [0000-0001-6373-8326](https://orcid.org/0000-0001-6373-8326)), Matthieu Bressac^3^ ([matthieu.bressac@imev-mer.fr](mailto:matthieu.bressac@imev-mer.fr); ORCID: 0000-0003-3075-3137), Emmanuelle Uher^3^ ([emmanuelle.uher@imev-mer.fr](mailto:emmanuelle.uher@imev-mer.fr); ORCID: 0000-0002-3379-0040), Maryline Montanes^3^ ([maryline.montanes@imev-mer.fr](mailto:maryline.montanes@imev-mer.fr); ORCID: 0000-0002-8796-849X) Elvira Pulido-Villena^5^ ([elvira.pulido@mio.osupytheas.fr](mailto:elvira.pulido@mio.osupytheas.fr); ORCID: 0000-0002-5436-2133), Cristina Santín^6,7^ ([c.santin@csic.es](mailto:c.santin@csic.es); ORCID: 0000-0001-9901-2658), Kahina Djaoudi^8^ ([kahina.djaoudi@septentrion-env.com](mailto:kahina.djaoudi@septentrion-env.com); ORCID: [0000-0002-7819-7304](https://orcid.org/0000-0002-7819-7304)), Pierre E. Galand^2^ ([galand@obs-banyuls.fr](mailto:galand@obs-banyuls.fr); ORCID: 0000-0002-2238-3247) Eva Ortega-Retuerta^1^ ([ortega-retuerta@obs-banyuls.fr](mailto:ortega-retuerta@obs-banyuls.fr); ORCID: [0000-0003-0780-8347](https://orcid.org/0000-0003-0780-8347))

1) Sorbonne Université, CNRS, Laboratoire d'Océanographie Microbienne (LOMIC), Observatoire Océanologique de Banyuls, Banyuls-sur-Mer, France

2) Sorbonne Université, CNRS, Laboratoire d’Ecogéochimie des Environnements Benthiques (LECOB), Observatoire Océanologique de Banyuls, Banyuls-sur-Mer, France

3) Sorbonne Université, CNRS, Laboratoire d'Océanographie de Villefranche (LOV), 06230 Villefranche-sur-Mer, France

4) Barcelona Supercomputing Center, Barcelona, Spain

5) IRD, CNRS, Mediterranean Institute of Oceanography (MIO), Aix-Marseille Université, Université de Toulon, Marseille, France

6) Biodiversity Research Institute (IMIB), CSIC – Universidad de Oviedo – Principality of Asturias, Mieres, 33600, Spain.

7) Biosciences Department, Swansea University, Swansea, SA2 8PP, UK.

8) Septentrion Environnement, Campus Nature Provence, Marseille, France.

**
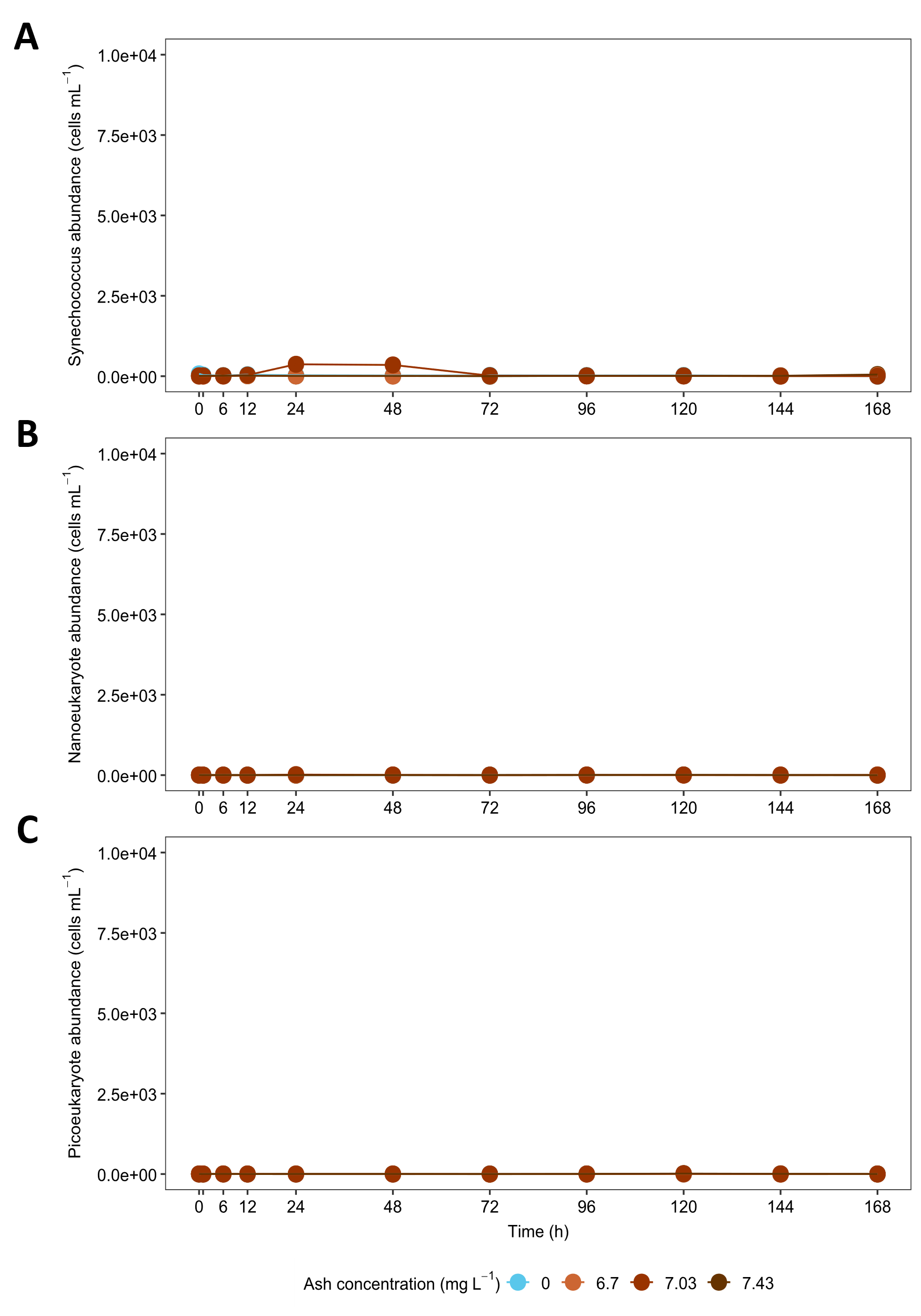
**

**Figure S1** Abundance of *Synechococcus sp.* (A), nanoeukaryotes (B) and picoeukaryotes (C) in “Bacterioplankton only” minicosms with different ash concentrations. The control value at 144h was considered as an outlier due to sample marking issues, and removed from the dataset.

**
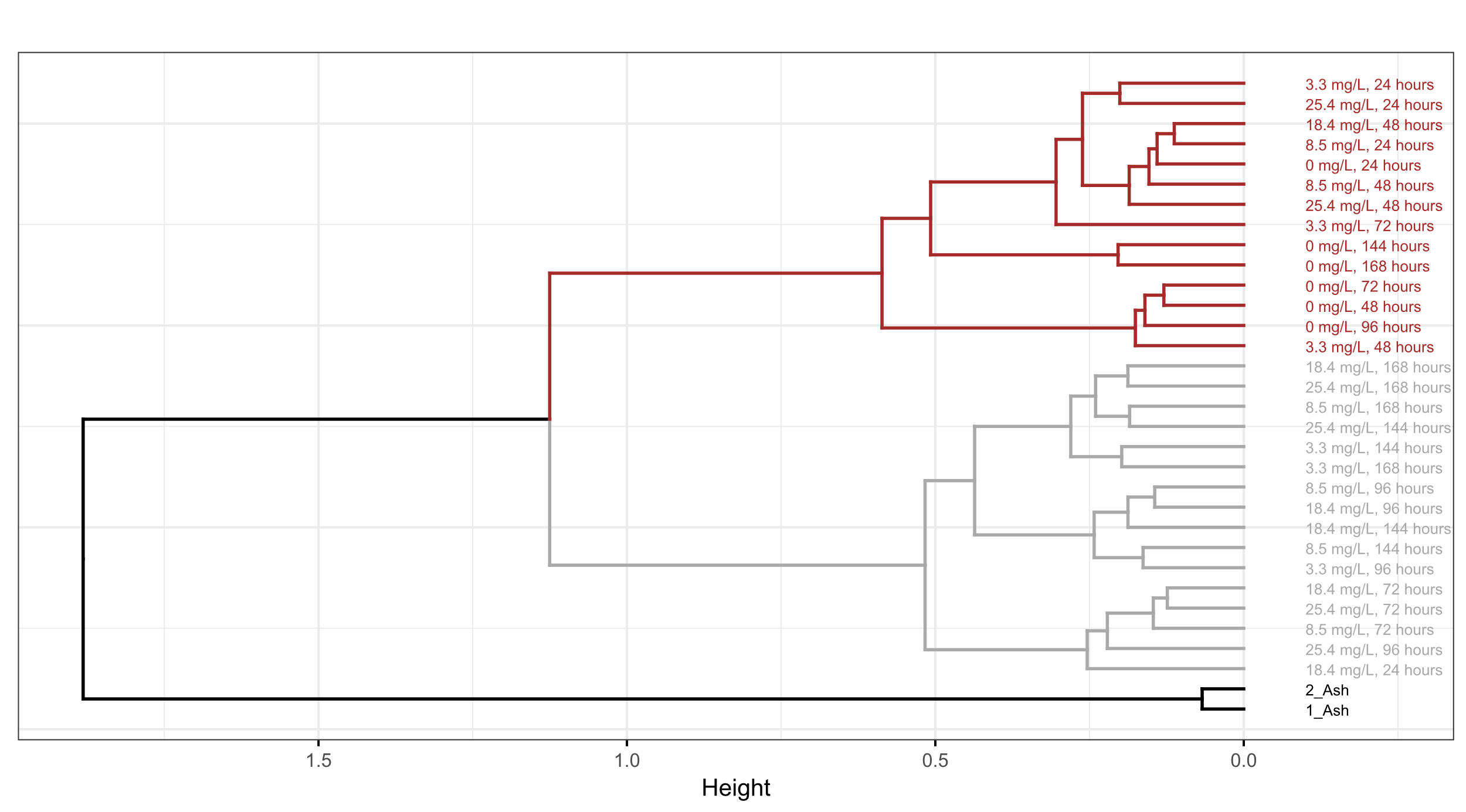
Figure S2** Dendrogram based on prokaryotic community composition (ASV level) using Ward’s hierarchical clustering method on a Bray-Curtis dissimilarity matrix. Different colours represent the defined clusters.


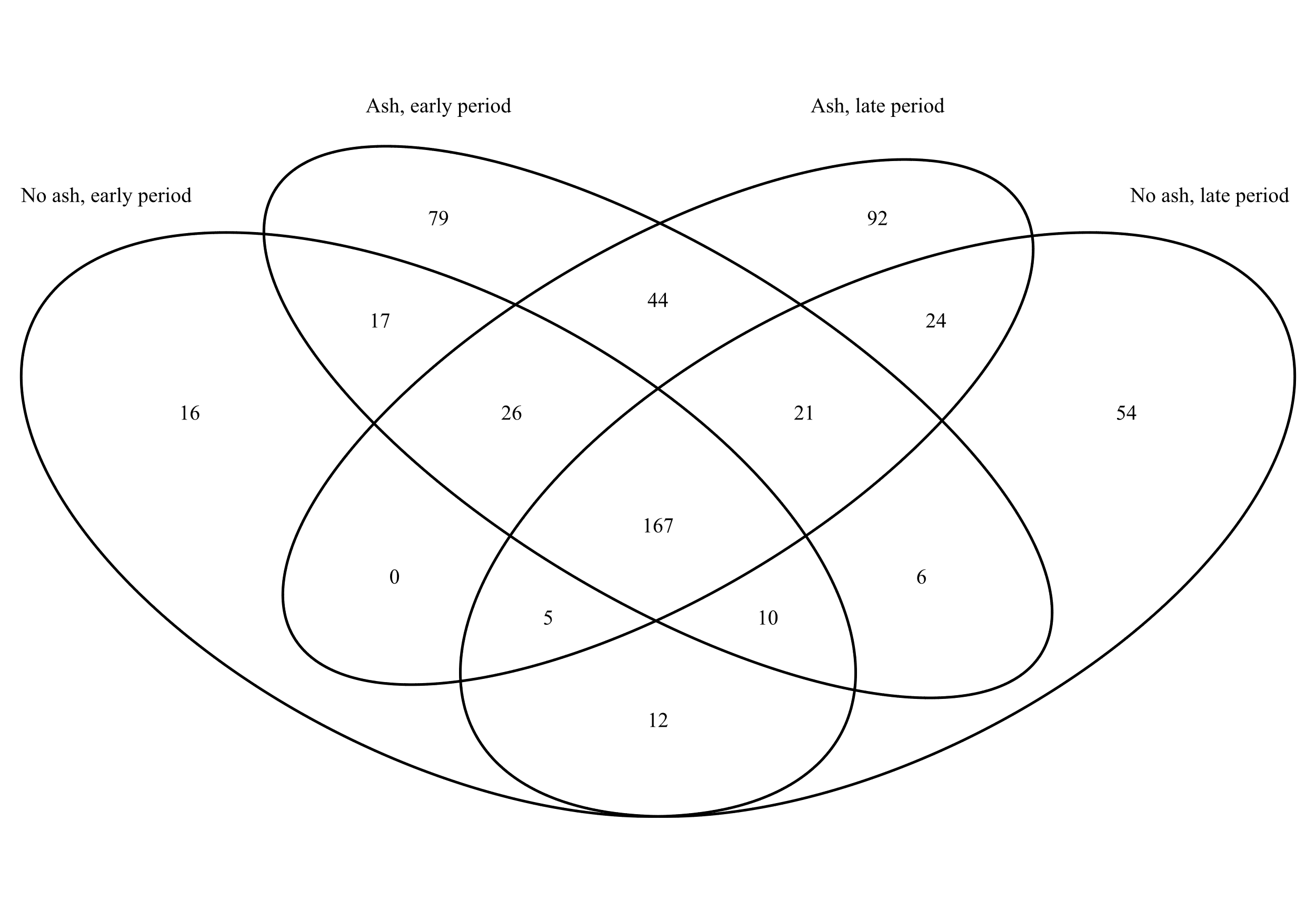


**Figure S3** Venn diagram illustrating the number of shared and unique ASVs between different experimental conditions. “Ash” refers to all ash-amended minicosms, regardless of the concentration.


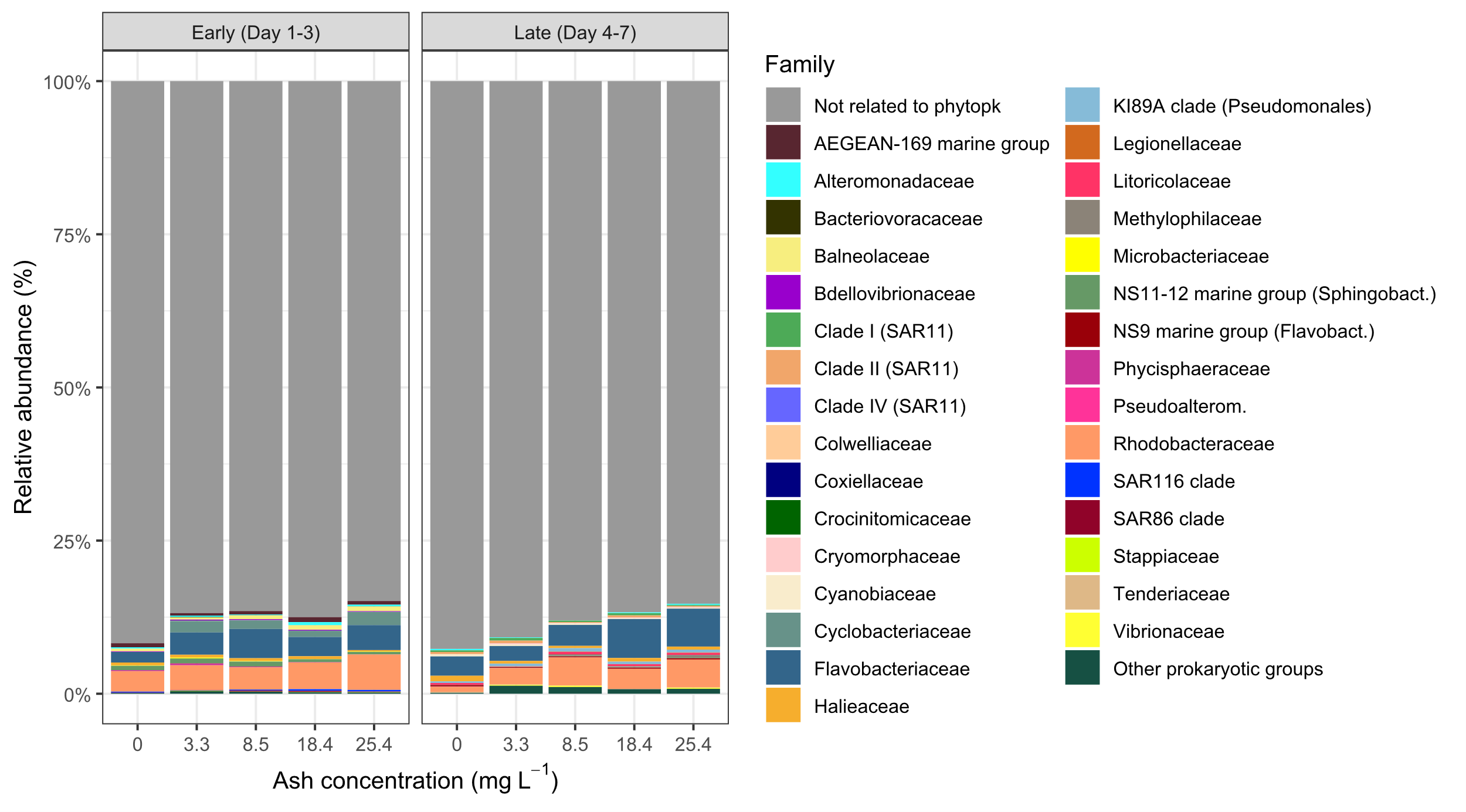


**Figure S4** Relative abundances of the prokaryotic ASVs significantly correlated with chlorophyll *a* levels, grouped by family, across experimental conditions during both the early and the late period of the experiment. “Other prokaryotic groups” represent the ASVs which were not assigned at the family level. “Not related to phytopk” refers to all ASVs that were not significantly correlated to chlorophyll *a* concentration.


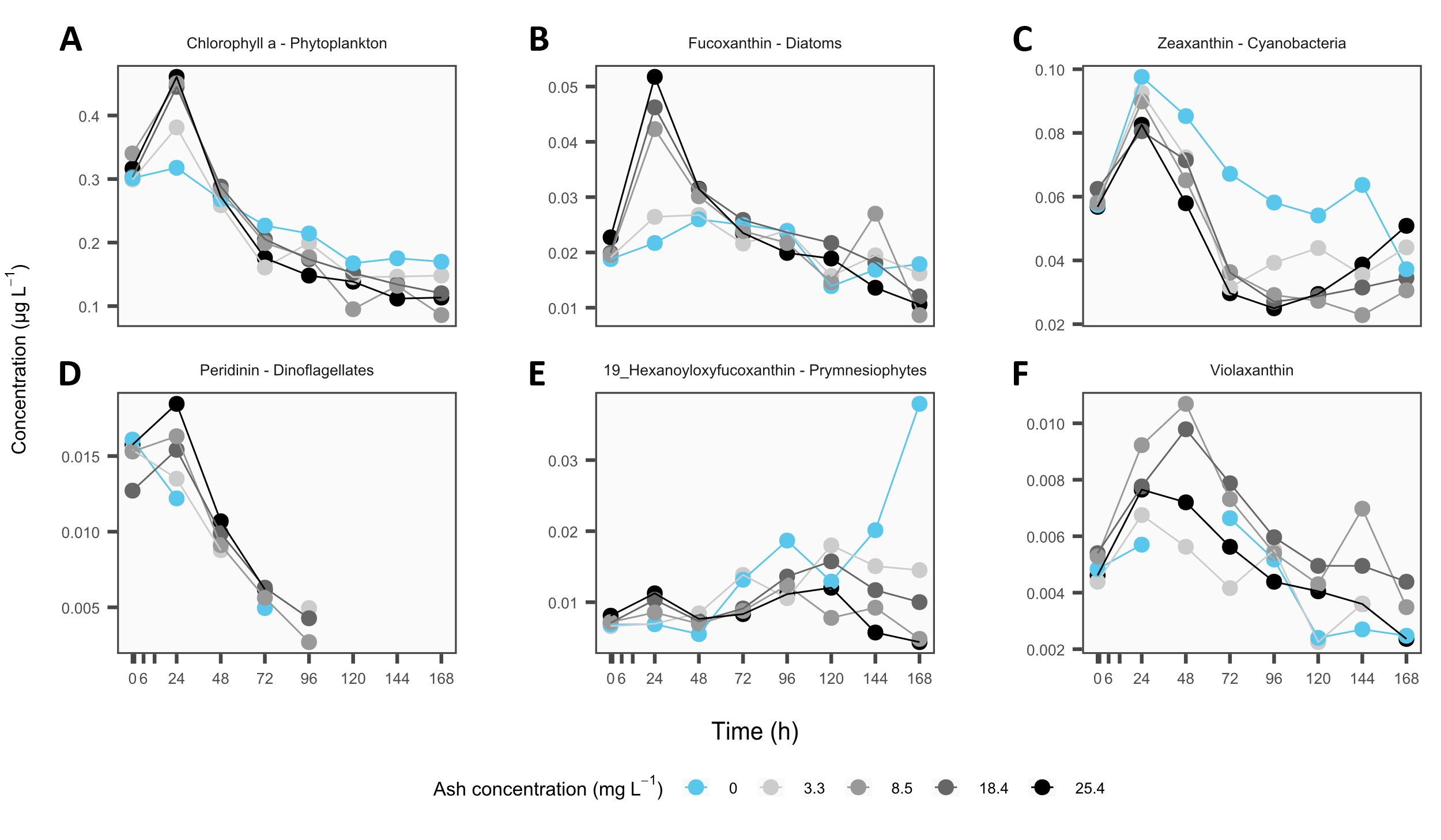


**Figure S5** Concentration of chlorophyll *a* (A), fucoxanthin, characteristic pigment of diatoms (B), zeaxanthin, characteristic pigment of cyanobacteria (C), peridinin, characteristic pigment of dinoflagellates (D), ^19^Hexanoyloxyfucoxanthin, characteristic pigment of prymnesiophytes (E) and Violaxanthin (F) through time in the “Plankton Community” minicosms with varying concentrations of ash.


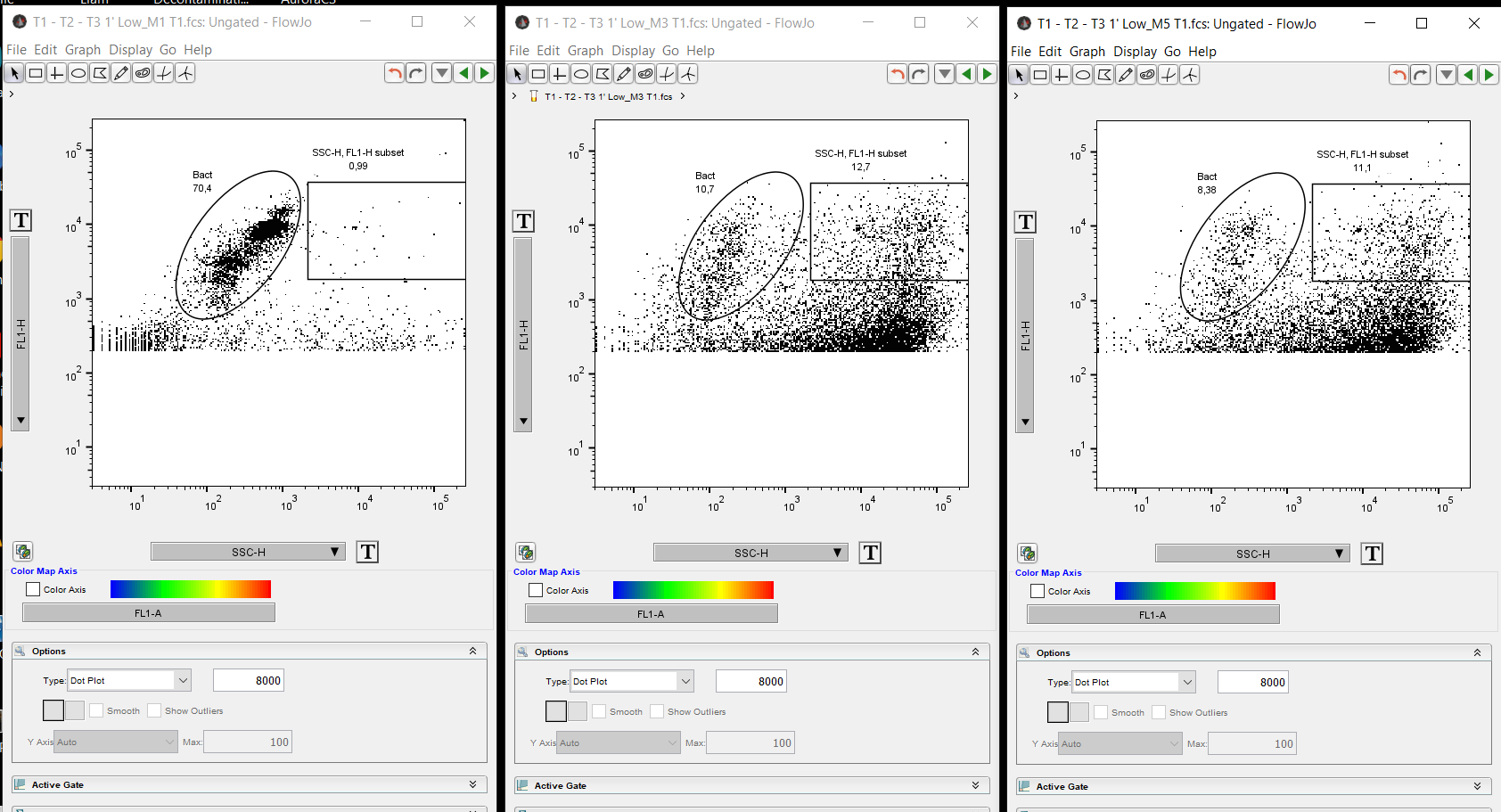


**A**

**B**

**Figure S6** Examples of cytograms of Side-scatter (SSC-H) versus green fluorescence intensity (FL1-H) showing prokaryotes stained with SYBR-green taken 1h after of incubation in the “bacterioplankton only” ash-free (A) and ash-amended (24.7 mg L^-1^; B) minicosms. Heterotrophic prokaryotes are in the ellipse, while the rectangle contains bigger and potentially particle-attached prokaryotes.

**Supplementary methods –** Nault et *al*., 2026: From flames to the ocean: biomass burning aerosols are associated with changes in prokaryotic communities in the Mediterranean Sea

***Dissolved iron and other trace metals***

Dissolved trace metals concentrations were measured at each sampling point in all minicosms. Samples were directly filtered through a 0.2 µm filter cartridge (Sartorius Sartobran P capsules 0.45/0.2-µm) connected to the sampling tube of the minicosms. Samples were collected into acid-cleaned LDPE bottles (1 for iron and 1 for all other trace metals), and acidified to pH 1.8 using ultrapure HCl. All trace metals, except iron, were then analysed using matrix separation and analyte pre-concentration with the commercially available seaFAST-pico™ (Elemental Scientific Inc., USA) system. Trace metals were then analysed after external calibration with ICAP TQ ICP-MS (Thermofisher, USA) in the eluted fractions obtained after SeaFAST procedure.

Dissolved iron measurements were carried out using the Flow Injection Analysis (FIA) method with chemiluminescence detection, as fully described in Bressac *et al.* (2021).

***Inorganic nutrients***

Nutrients samples were taken in all minicosms at each sampling point. Total Dissolved Phosphorus (TDP) and Total Dissolved Nitrogen (TDN) were analysed using a segmented flow analyser after high-temperature (120°C) persulfate wet oxidation mineralization (Pujo-Pay and Raimbault, 1994). Particulate phosphorus (PP) was analysed using a segmented flow analyser (AAIII HR Seal Analytical) according to Aminot et Kérouel (2007) after conversion to phosphate through a wet oxidation method based on a persulfate digestion at 120 °C (Raimbault *et al.*, 1999). The limit of detection, determined by analysing 10 blank filters, was 1 nmol L^-1^.

***Fluorescent dissolved organic matter***

FDOM samples were filtered through pre-combusted glass fiber filters (GF/F; Whatman USA) and stored in acid-washed HDPE bottles at -20°C. Before analysis, samples were thawed at room temperature. Three-dimensional fluorescence excitation-emission matrices (EEMs) were measured using a JASCO FP-8500 spectrofluorometer, with excitation wavelengths from 250 to 450 nm (5 nm increments) and emission wavelengths from 250 to 560 nm (2 nm increments) and a scan speed of 5000 nm.min^-1^. Milli-Q EEMs were measured in every batch of analysis and used as blanks. Data were processed using the drEEM toolbox (v0.6.6; (Murphy *et al.*, 2013). First, the matrices were cleaned by removing Raman and Rayleigh scatter and noisy signals. Due to low absorbance coefficients in the measured samples (all minicosms at T0 and after 144h), inner filter effect correction was not needed (Hur *et al.*, 2008). Data were normalized to Raman Units (RU) using the area under the Milli-Q water Raman peak at 350 nm (Lawaetz and Stedmon, 2009).

Parallel Factor Analysis (PARAFAC) was performed in MATLAB (vR2023b, MathWorks, Natick, MA, USA) to characterise the nature of fluorophores in the samples, following the approach of Murphy *et al.* (2013). A four-component model was developed with the “non-negativity” criterion on 132 EEMs (8 outliers removed), and validated with a visual inspection of the residuals, split-half analysis and percentage of explained variance (99.7%). To classify fluorophores types (humic-like, protein-like), the excitation and emission spectra of the identified components were compared to over 280 published components identified during previous studies in the Openfluor database, as detailed in Murphy *et al.* (2014). The fluorescence signal was decomposed into four distinct components: C_1_ (excitation 250nm / emission 408nm, terrestrial humic-like DOM), C_2_ (excitation 265nm / emission 460nm, humic-like DOM), C_3_ (excitation 265nm / emission 322nm, protein/amino-acid-like DOM), C_4_ (excitation 285nm / emission 340nm, protein-like DOM). Two components (C_1_ and C_4_) were significantly related to ash concentration (Pearson correlation analysis, p < 0.05), and therefore kept for statistical analyses.

***Particulate organic carbon***

Samples were taken at the onset (0h) of the experiment, and daily from day 1 to day 7. Samples of 2.7 L were filtered through pre-combusted (450°C, 4 h) and pre-weighed GF/F filters. Filters were then stored in petri dishes and dried at 60°C for 24 h. Shortly before analysis, each sample was acidified by adding HCl (2 N) to remove the inorganic carbon fraction. POC concentrations were measured by an elemental analyser (EA-IRMS; Vario Pyrocube, Elementar®).

***Phytoplankton pigments***

Samples for pigment analyses were taken at the same intervals as the DNA samples, in the “plankton community” minicosms only. Samples of 2.7 L were filtered through GF/F filters and store frozen until analysis (-80°C). The filters were extracted using 3 mL of methanol (100%), crushed by sonication and clarified for one hour by vacuum filtration. The extracts were analysed using a high-performance liquid chromatography (HPLC) system (Agilent Technologies 1200 series). The pigments were separated and quantified as described in Ras *et* *al.* (2008).

**REFERENCES**

Aminot, A. and Kérouel, R. (2007) *Dosage automatique des nutriments dans les eaux marines: méthodes en flux continu*. Editions Quae.

Bressac, M. *et al.* (2021) ‘Subsurface iron accumulation and rapid aluminum removal in the Mediterranean following African dust deposition’, *Biogeosciences*, 18(24), pp. 6435–6453. Available at: https://doi.org/10.5194/bg-18-6435-2021.

Hur, J., Hwang, S.-J. and Shin, J.-K. (2008) ‘Using Synchronous Fluorescence Technique as a Water Quality Monitoring Tool for an Urban River’, *Water, Air, and Soil Pollution*, 191(1), pp. 231–243. Available at: https://doi.org/10.1007/s11270-008-9620-4.

Lawaetz, A.J. and Stedmon, C.A. (2009) ‘Fluorescence Intensity Calibration Using the Raman Scatter Peak of Water’, *Applied Spectroscopy*, 63(8), pp. 936–940. Available at: https://doi.org/10.1366/000370209788964548.

Murphy, K.R. *et al.* (2013) ‘Fluorescence spectroscopy and multi-way techniques. PARAFAC’, *Analytical Methods*, 5(23), pp. 6557–6566. Available at: https://doi.org/10.1039/C3AY41160E.

Murphy, K.R. *et al.* (2014) ‘OpenFluor– an online spectral library of auto-fluorescence by organic compounds in the environment’, *Analytical Methods*, 6(3), pp. 658–661. Available at: https://doi.org/10.1039/C3AY41935E.

Pujo-Pay, M. and Raimbault, P. (1994) ‘improvement of the wet-oxidation procedure for simultaneous determination of particulate organic nitrogen and phosphorus collected on filters’, *Marine Ecology Progress Series*, 105, pp. 203–207. Available at: https://doi.org/10.3354/meps105203.

Raimbault, P. *et al.* (1999) ‘Wet-oxidation and automated colorimetry for simultaneous determination of organic carbon, nitrogen and phosphorus dissolved in seawater’, *Marine Chemistry*, 66(3), pp. 161–169. Available at: https://doi.org/10.1016/S0304-4203(99)00038-9.

Ras, J., Claustre, H. and Uitz, J. (2008) ‘Spatial variability of phytoplankton pigment distributions in the Subtropical South Pacific Ocean: comparison between in situ and predicted data’, *Biogeosciences*, 5(2), pp. 353–369. Available at: https://doi.org/10.5194/bg-5-353-2008.
